# Arterial stiffness in long-term breast cancer survivors: a propensity score–matched analysis in primary prevention

**DOI:** 10.1186/s40959-026-00476-0

**Published:** 2026-03-26

**Authors:** Renzo Melchiori, Miguel Rizzo, Noelia Brenzoni, Hector Gonzalez Aleman, Pamela Alarcon, Guido Garcia, Alejandro Hita, Sergio Gonzalez

**Affiliations:** 1 Unit of Cardiometabolism, Department of Cardiology, Hospital Universitario Austral, Buenos Aires, Argentina; 2 Unit of Cardiooncology, Department of Cardiology, Hospital Universitario Austral, Buenos Aires, Argentina; 3 Cancer Immunobiology Laboratory, Instituto de Investigaciones en Medicina Traslacional, Universidad Austral-Consejo Nacional de Investigaciones Cientificas y Tecnologicas (CONICET), Buenos Aires, Argentina; 4 Department of Clinical Oncology, Hospital Universitario Austral, Derqui-Pilar, Buenos Aires, Argentina

**Keywords:** Breast cancer survivors, Pulse wave velocity, Arterial stiffness, Cardio-oncology

## Abstract

**Background:**

Breast cancer treatment may be associated with adverse cardiovascular effects. Pulse wave velocity (PWV) is a marker of arterial stiffness and a validated predictor of cardiovascular events. The aim of this study was to evaluate whether a history of breast cancer (HBC), beyond the first year after diagnosis, is associated with higher PWV in middle-aged women in a primary prevention setting.

**Methods:**

Women aged 35 to 65 years evaluated at a cardiometabolic unit between September 2021 and March 2024 were included if valid PWV measurements obtained by automated oscillometry were available. Exclusion criteria comprised previous cardiovascular disease, chronic kidney disease stage > II, and inflammatory conditions. Participants were categorized according to HBC, defined as a diagnosis made at least one year prior to evaluation. A propensity score adjusted for age, hypertension, dyslipidemia, smoking status, physical inactivity, diabetes, obesity and use of statins was constructed, and 1:1 matching was performed. PWV (m/s) was the primary outcome. In addition, inverse probability weighting (IPW) analysis was conducted.

**Results:**

A total of 2,318 women were analyzed (mean age 51.0 ± 7.3 years); 53 (2.3%) had HBC, with a median time of 67 months since diagnosis. PWV was significantly higher in women with HBC compared with those without cancer (7.85 ± 0.99 vs. 7.34 ± 1.09 m/s; *p* = 0.0008), with no significant differences in traditional cardiovascular risk factors. After matching, HBC was associated with a 0.34 m/s increase in PWV (95% CI: 0.24–0.45; *p* < 0.001). IPW analysis confirmed these findings.

**Conclusions:**

A history of breast cancer is associated with increased arterial stiffness independently of traditional risk factors, underscoring the relevance of vascular surveillance in breast cancer survivors.

**Supplementary Information:**

The online version contains supplementary material available at 10.1186/s40959-026-00476-0.

## Introduction

Cancer remains one of the leading causes of global morbidity and mortality, with approximately 17 million new cases diagnosed annually [1]. Despite substantial advances in oncologic therapies, cardiovascular disease has emerged as a competing cause of death among cancer survivors. This association is multifactorial and likely driven by shared cardiovascular risk factors, systemic inflammation, and treatment-related vascular toxicity [2–4]. Recent studies have proposed a prior cancer diagnosis as an independent cardiovascular risk factor in secondary prevention settings, in which conventional preventive strategies have failed to fully mitigate the excess risk observed in this population [5].

Breast cancer treatment commonly involves chemotherapy, endocrine therapy, anti Her therapy and radiotherapy, both of which can induce endothelial injury and accelerate vascular remodeling, leading to increased arterial stiffness during active treatment [6]. Pulse wave velocity (PWV), a robust marker of central arterial stiffness, has been consistently associated with cardiovascular [7], cerebrovascular [8], and renal outcomes [9].

Emerging evidence indicates that these vascular alterations may persist long after completion of cancer therapy, suggesting premature vascular aging even among clinically stable survivors [10]. However, data addressing arterial stiffness in survivors beyond the early post-treatment phase and in primary prevention settings remain limited. Therefore, the present study aimed to evaluate whether a history of breast cancer, beyond one year after diagnosis, is independently associated with increased arterial stiffness, assessed by oscillometric PWV measurement, in middle-aged women without overt cardiovascular disease.

## Methods

### Study design and population

This cross-sectional study was conducted using an anonymized registry of women enrolled in a Cardiovascular Prevention Program at Hospital Universitario Austral (CARFARE; ClinicalTrials.gov identifier NCT04040777) between September 2021 and September 2024. Participants were enrolled after providing written informed consent. The registry includes individuals undergoing both primary and secondary prevention. In the primary prevention setting, individuals presented with traditional cardiovascular risk factors (hypertension, dyslipidemia, smoking, obesity, sedentary lifestyle, and diabetes) and/or suspected subclinical atherosclerosis requiring risk reclassification.

The objectives of the program were to assess the presence of subclinical atherosclerosis, refine cardiovascular risk stratification, and optimize preventive interventions, including pharmacologic therapy, exercise prescription, and dietary modification.

### Study objective

The primary objective was to assess whether a remote history of breast cancer is associated with increased arterial stiffness, measured by pulse wave velocity (PWV), in middle-aged women without prior cardiovascular disease, after adjustment for traditional cardiovascular risk factors using propensity score–based methods.

### Inclusion and exclusion criteria

Women aged 35–65 years in primary prevention with valid PWV measurements obtained using an oscillometric device validated against intra-arterial measurements were eligible [11, 12].

Exclusion criteria included prior cardiovascular or cerebrovascular events (acute myocardial infarction, coronary artery bypass grafting, percutaneous coronary intervention, or stroke), heart failure, chronic kidney disease stage III or higher, inflammatory or rheumatologic disorders, cancer diagnosed less than one year before enrollment, or active oncologic treatment other than hormone therapy. Patients receiving chemotherapy or immunotherapy were excluded.

### Exposure definition

Exposure was defined as a history of breast cancer diagnosed at least one year before enrollment in the prevention program. Among patients receiving active oncologic treatment, only hormone therapy was permitted. Participants were classified into two groups: women without a history of breast cancer (control group) and women with a history of breast cancer (breast cancer group).

### Clinical assessment

All participants underwent a standardized clinical evaluation, including medical history, physical examination, and blood pressure and heart rate measurements. Traditional cardiovascular risk factors—smoking status, diabetes mellitus, dyslipidemia, obesity, and physical inactivity—were systematically assessed using a standardized questionnaire. Current medications were recorded.

### Arterial stiffness measurement

PWV was assessed at the left brachial artery using the Mobil-O-Graph® device, which estimates central arterial stiffness through pulse wave analysis and a validated waveform amplification algorithm.

### Statistical analysis

Continuous variables are presented as mean ± standard deviation and categorical variables as frequencies and percentages. Between-group comparisons were performed using unpaired t-tests or Mann–Whitney U tests for continuous variables and chi-square tests for categorical variables.

To estimate the independent association between breast cancer history and PWV, two complementary causal inference approaches were applied: propensity score matching (PSM) and inverse probability weighting (IPW).

For PSM, propensity scores were estimated using logistic regression including age, hypertension, smoking, dyslipidemia, diabetes, physical inactivity, obesity, and use of statins, followed by 1:1 nearest-neighbor matching. Covariate balance was assessed using standardized mean differences. The average treatment effect (ATE) was estimated as the adjusted difference in PWV between groups.

For IPW, stabilized inverse probability weights were calculated from the same propensity score model, and the ATE was estimated using robust standard errors.

**Fig. 1:**
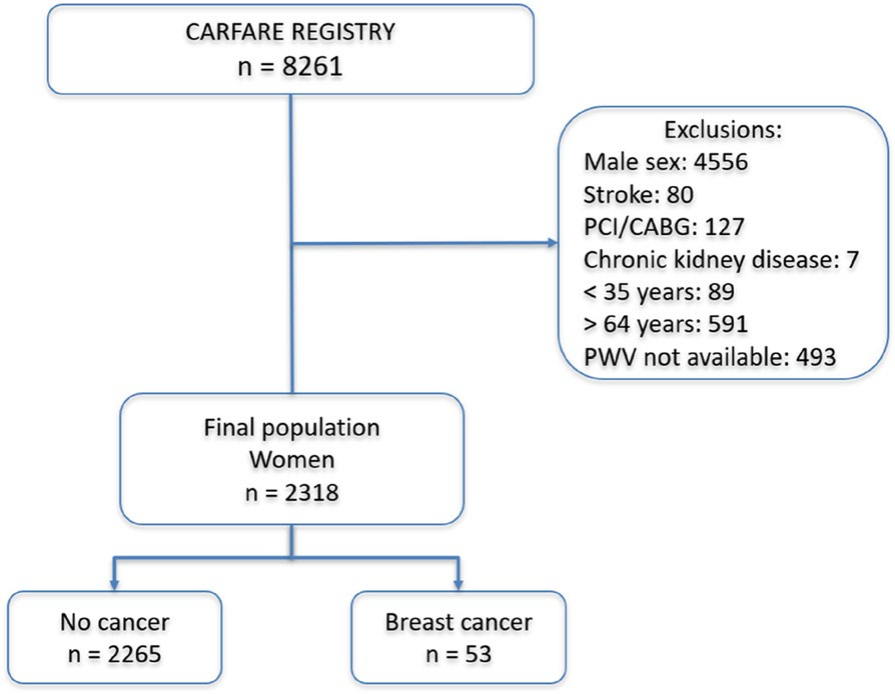
Flow chart

A two-sided *p* value < 0.05 was considered statistically significant. All analyses were performed using Stata BE version 17.0 (Fig. 1).

## Results

A total of 2,318 women aged 35 to 65 years, with no prior cardiovascular history, were included. Among them, 53 were diagnosed with breast cancer and 2,265 served as controls. The oncologic group was significantly older (54.5 ± 6.6 vs. 50.9 ± 7.3 years; *p* = 0.0003) and had a slightly higher heart rate (71.3 ± 14.0 vs. 67.8 ± 11.1 bpm; *p* = 0.024). See Table 1.

**Table 1:** Baseline characteristics

Variable	No Breast Cancer (*n* = 2265)	Breast Cancer (*n* = 53)	*p*-value
Age (years)	50.9 ± 7.3	54.5 ± 6.6	0.0003
PWV (m/s)	7.34 ± 1.09	7.85 ± 0.99	0.0008
Heart rate (beats/min)	67.8 ± 11.1	71.3 ± 14.0	0.02
Systolic BP (mmHg)	122.7 ± 15.9	125.4 ± 16.4	0.22
Diastolic BP (mmHg)	77.7 ± 10.5	79.8 ± 10.9	0.16
Hypertension (%)	24.6	18.9	0.34
Smoking (%)	12.3	11.3	0.83
Dyslipidemia (%)	36.5	34.0	0.70
Sedentarism (%)	28.8	37.7	0.16
Diabetes (%)	6.3	5.7	0.85
Obesity (%)	14.6	20.7	0.21
Medication class			
ACE inhibitors	103 (4.5%)	1 (1.9%)	0.26
BRAT1	289 (12.7%)	8 (15.1%)	0.68
Thiazide diuretics	97 (4.3%)	2 (3.8%)	0.67
Antialdosteronics	8 (0.4%)	0 (0%)	0.64
Alpha-blockers	3 (0.1%)	0 (0%)	0.79
Statins	485 (21.4%)	9 (17.0%)	0.48
Hormonal therapy		16 (30.1%)	-

The median time from cancer diagnosis to enrollment in the primary prevention registry was 67.5 months (IQR 25–75: 29.5–152.5). Sixty-six percent of patients had TNM stage IA disease. Overall, 58% of the oncologic cohort received chemotherapy, 61.7% received radiotherapy, and 78% received hormone therapy. At the time of cohort entry, 30% of patients were still receiving ongoing hormonal treatment. The specific endocrine therapies administered were tamoxifen in 73.2% of patients (n = 30), aromatase inhibitors in 24.4% (n = 10), and leuprolide in 2.4% (n = 1). Importantly, among the 35 patients with stage IA disease, only one underwent surgery alone, whereas all others received at least one form of systemic therapy (chemotherapy and/or endocrine therapy). The remaining oncologic characteristics are presented in Table 2. Pulse wave velocity was higher in patients with breast cancer (7.85 ± 0.99 vs. 7.34 ± 1.09 m/s; *p* = 0.0008).

**Table 2:** Oncological characteristics of breast cancer survivors (*n* = 53)

Characteristic	**n (%)**
Time since diagnosis (months), median [IQR]	67.5 [29.5–152.5]
TNM Stage	
• Stage 0	2 (3.8)
• Stage I	35 (66.0)
• Stage IIA	7 (13.2)
• Stage IIB	7 (13.2)
• Stage IIIA	1 (1.9)
• Stage IIIC	1 (1.9)
Treatments	
• Chemotherapy	32 (57.1)
• Radiotherapy	33 (61.8)
• Hormonal therapy	41 (78.0)
Tamoxifen	30 (73.2%)
Aromatase inhibitors	10 (24.4%)
Leuprolide	1 (2.4%)
Chemotherapy regimens	
• None	23 (43.4)
• CMF/variants	2 (3.8)
• AC-based	21 (39.6)
• Taxanes + anti-HER2	4 (7.6)
• Anti-HER2 triple-therapy	2(3.8)

*AC* indicates anthracycline–cyclophosphamide–based chemotherapy, *CMF* indicates cyclophosphamide, methotrexate, and 5-fluorouracil

### PSM model results

The logistic regression model used to estimate the propensity score included age, obesity, smoking status, dyslipidemia, physical inactivity, hypertension, statin therapy, and diabetes (Table 3). Age was independently associated with treatment assignment (β = 0.088; *p* < 0.001), while the remaining covariates were retained based on clinical relevance. After 1:1 nearest-neighbor matching, covariate balance substantially improved, with a reduction in mean standardized bias from 17.1% to 5.4% and a decrease in pseudo-R^2^ from 0.056 to 0.014. All standardized mean differences were below 0.10 (Supplementary Figs. 1–2). In the matched sample, the average treatment effect (ATE) showed that women with a history of breast cancer had a pulse wave velocity (PWV) 0.34 m/s higher than controls (95% CI 0.24–0.45; *p* < 0.001).

**Table 3:** Covariate balance before and after 1:1 propensity score matching

Covariate	Breast Cancer (Unmatched)	Control (Unmatched)	Std. Bias Before (%)	Breast Cancer (Matched)	Control (Matched)	Std. Bias After (%)
Dyslipidemia	0.3396	0.3651	−5.3	0.3396	0.2642	15.7
Smoking	0.1132	0.1227	−2.9	0.1132	0.1132	0.0
Hypertension	0.1887	0.2459	−13.9	0.1887	0.1887	0.0
Sedentary	0.3774	0.2883	18.9	0.3774	0.4340	−12.0
Diabetes	0.0566	0.0631	−2.7	0.0566	0.0377	7.9
Obesity	0.2076	0.1457	16.2	0.2076	0.1887	4.9
Statins	0.1132	0.2004	−24.1	0.1132	0.1132	0.0
Age (years)	54.53	50.87	52.6	54.53	54.34	2.7

### IPW model results

The propensity score model included the same clinical covariates as in the PSM analysis. Stabilized inverse probability of treatment weights were estimated, and covariate balance was assessed using standardized mean differences, all of which were < 0.10 after weighting. The distribution of stabilized weights was appropriate (mean 1.00; range 0.35–5.12), without evidence of extreme values.

**Fig. 2:**
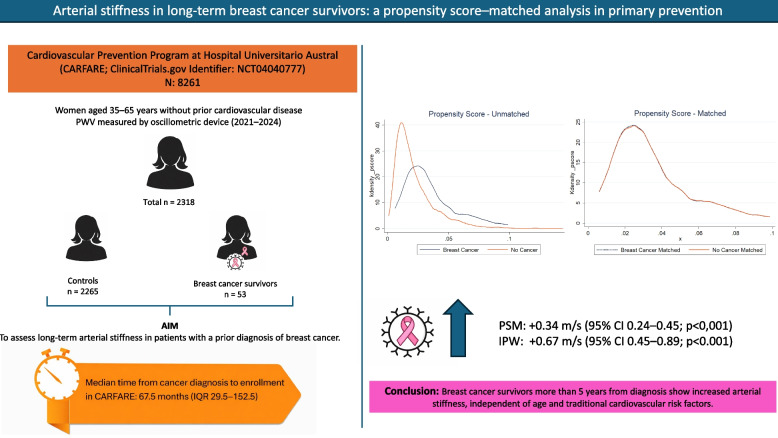
Central illustration

Among 2,318 individuals included in the weighted analysis, the average treatment effect (ATE) estimated using IPW showed that a history of breast cancer was associated with a significant increase in PWV (ATE = 0.67 m/s; 95% CI 0.45–0.89; *p* < 0.001) Table 4. Robust standard errors were used for variance estimation (Fig. 2).

**Table 4:** Association between history of breast cancer and pulse wave velocity: propensity score–based analyses

Method	Estimand	N	Effect Estimate (m/s)	95% CI	*p*-value
Propensity Score Matching (1:1, logit)	ATE	2318	0.34	0.24–0.45	< 0.001
IPW	ATE	2318	0.67	0.45–0.89	< 0.001

*ATE* Average Treatment Effect, *IPW* inverse probability weighted

## Discussion

In this observational study, we found that women with a history of breast cancer exhibit a persistent increase in arterial stiffness more than five years (median 67 months) after completion of cancer treatment, whether systemic or locoregional, even in the absence of active disease. After rigorous adjustment for traditional cardiovascular risk factors and incorporating baseline statin therapy into the propensity score model, breast cancer survivorship remained independently associated with higher PWV, with an average treatment effect of 0.34 m/s. The inclusion of statin therapy was specifically justified by emerging evidence suggesting that statins may attenuate aortic stiffness during anthracycline-based chemotherapy, as recently demonstrated in a randomized clinical trial [13]. The magnitude of the observed effect is clinically relevant and suggests that breast cancer and/or its therapies may leave a durable vascular footprint beyond the active treatment phase [14].

A distinctive feature of our work is the analytical approach oriented toward causal inference. The complementary use of inverse probability weighting (IPW) and propensity score matching (PSM) allowed independent balancing of covariate distributions between exposed and unexposed groups, in line with current methodological recommendations for observational studies. These two strategies, with different assumptions and sources of bias, converged on very similar results, increasing the robustness of the observed association and reducing the likelihood of residual confounding.

It is important to emphasize that women included as controls in the CARFARE registry were not healthy individuals free of cardiovascular risk factors, but rather patients referred for structured primary prevention due to the presence of traditional cardiovascular risk factors and/or suspected subclinical atherosclerosis requiring risk reclassification and therapeutic optimization. As a result, baseline cardiovascular risk burden and preventive therapies were broadly comparable between groups. This context strengthens the internal validity of the findings, as the observed difference in arterial stiffness is unlikely to be explained by systematic differences in baseline cardiovascular risk.

Our findings add to the growing body of evidence showing excess cardiovascular risk among cancer survivors in general, and breast cancer survivors in particular, which is not fully explained by traditional risk factors [4, 15]. Previous studies have documented increases in arterial stiffness during or shortly after chemotherapy, radiotherapy, or targeted therapies, with follow-up typically limited to 6–18 months [3, 5, 6, 16, 17]. In contrast, our study provides information on arterial stiffness in the long-term survivorship phase, showing that increased PWV persists several years after completion of systemic therapy. This suggests that vascular remodeling associated with breast cancer and its treatments may not be merely a transient phenomenon, but potentially long-lasting.

Arterial stiffness is strongly dependent on age, systolic blood pressure, and other cardiovascular risk factors [2, 8, 10]. By focusing on a relatively young cohort with a low prevalence of comorbidities, we minimized the influence of these major determinants and were better able to isolate the independent contribution of oncological history. This design is consistent with population-based data showing stronger associations between cancer and arterial stiffness in younger individuals, whereas in older adults the dominant influence of chronological age and hypertension tends to attenuate effect size [2, 10].

Although mechanistic pathways cannot be directly inferred from the present observational design, several biologically plausible mechanisms may explain the observed association. The mechanisms that may explain the sustained increase in PWV among breast cancer survivors are multifactorial. On one hand, traditional cardiovascular risk factors—hypertension, dyslipidemia, central obesity, and glycemic disorders—promote extracellular matrix remodeling, medial calcification, and loss of elastic fibers [8, 10]. Superimposed on this substrate, cardiometabolic alterations and chronic low-grade inflammation, common in oncological populations, contribute to endothelial dysfunction, increased oxidative stress, and activation of profibrotic pathways [17–20]. On the other hand, therapies such as anthracyclines, breast radiotherapy, and antiangiogenic agents are associated with direct vascular damage and measurable increases in arterial stiffness both in the acute phase and long term [3, 5, 6, 21–24]. It is likely that the interaction among these pathways—baseline risk factors, cardiometabolic disruption, persistent inflammation, and treatment-related vascular toxicity—explains the magnitude and persistence of the PWV increase observed in our cohort.

In our cohort, approximately 40% of breast cancer survivors had been exposed to anthracyclines, agents consistently associated with increased arterial stiffness and endothelial dysfunction in both short- and long-term follow-up studies. Although our study was not sufficiently powered to perform treatment-specific analyses, anthracycline exposure may have contributed to the observed increase in PWV.

Additionally, 78% of survivors received endocrine therapy, predominantly tamoxifen. This agent has been associated in some studies with potentially favorable vascular effects, which may partially attenuate cardiovascular risk. In contrast, a smaller proportion of patients receiving hormonal therapy were treated with aromatase inhibitors (24.4%) and leuprolide (2.4%), agents linked to a more pronounced estrogen-deprivation state and potentially higher cardiovascular risk. Although the present study was not designed to evaluate differences according to type of endocrine therapy, the high prevalence of hormonal exposure underscores the complexity of vascular determinants during the survivorship phase.

In this context, menopause is a recognized determinant of increased arterial stiffness; however, objective menopausal status was not available in our registry. As a proxy, we applied an age-based criterion (> 45 years), observing that the majority of women in both groups met this threshold, suggesting a comparable age distribution. Furthermore, the potential overlap among natural menopause, chemotherapy-induced ovarian failure, and endocrine therapy–related estrogen deprivation indicates that multiple converging mechanisms may contribute to vascular aging in this population.

Population-based evidence also suggests that the relationship between arterial stiffness and cancer may be bidirectional. In the Kailuan Study, elevated brachial–ankle PWV values were associated with higher cancer incidence and cancer-related mortality, raising the hypothesis that arterial stiffness may act as a marker and possible mediator of adverse outcomes [1]. Our results complement these findings by showing that once cancer has developed and been treated, arterial stiffness remains increased in the long term, potentially contributing to the excess cardiovascular events described among survivors [4, 15].

From a clinical perspective, PWV may represent an attractive marker for cardiovascular risk stratification in cardio-oncology among women with breast cancer. Recent studies indicate that arterial stiffness is associated with a higher likelihood of treatment-related cardiotoxicity, major cardiovascular events, and mortality in cancer patients [7, 16]. Moreover, estimated or directly measured PWV could be integrated into breast cancer–specific risk prediction models, complementing existing tools for assessing cardiotoxicity risk and long-term cardiovascular disease [25]. The observation that intensive lifestyle modifications are associated with lower arterial stiffness in cancer survivors [9] reinforces the concept that PWV is not only a marker of established damage but also a potential therapeutic target.

Taken together, our findings indicate that women with a history of breast cancer exhibit persistently higher arterial stiffness more than five years, on average, after completion of their initial cancer treatment, even when the disease is in remission and traditional risk factors are controlled. Although causality cannot be definitively established in a cross-sectional design, the consistency of the association across multiple adjustment strategies strengthens the robustness of the finding. These results add to the growing evidence supporting the potential value of PWV assessment in long-term follow-up of breast cancer survivors and underscore the importance of preventive strategies aimed at preserving cardiovascular health in this population.

### Limitations

Among the limitations of our study, the primary one is its observational cross-sectional design. Although advanced statistical methods, including propensity score–based approaches, were applied to mitigate confounding, residual confounding and reverse causality cannot be completely excluded. In particular, baseline (pre-cancer) PWV measurements were not available, preventing definitive conclusions regarding temporal directionality.

Another important limitation is the relatively small number of breast cancer survivors included in the matched analysis (*n* = 53), which limits statistical power for treatment-specific subgroup analyses. Therefore, our findings should be interpreted as reflecting a global survivorship effect rather than the impact of specific therapeutic modalities such as anthracyclines, radiotherapy, or endocrine therapy. Larger prospective studies will be required to disentangle therapy-specific vascular effects.

Another relevant limitation is the use of oscillometric devices to estimate PWV. The Mobil-O-Graph derives aortic PWV using a transfer function and a characteristic impedance model rather than direct carotid–femoral measurements. However, multiple validation studies [11, 12] have demonstrated good agreement, supporting its use in clinical and epidemiological research. This is particularly relevant in oncological cohorts, where noninvasive, reproducible, and operator-independent techniques are required.

Menopausal status was not directly available in the registry and may influence arterial stiffness. Although age was carefully accounted for in matching and weighting procedures, and age distributions were well balanced between groups, residual confounding related to menopausal transition or chemotherapy-induced ovarian failure cannot be completely excluded.

## Conclusions

Our study shows that women with a remote history of breast cancer exhibit significantly higher arterial stiffness independent of conventional cardiovascular risk factors. This finding may reflect persistent vascular remodeling associated with cancer survivorship. Further studies are needed to clarify the underlying mechanisms and to evaluate targeted strategies aimed at mitigating long-term cardiovascular risk in this growing population.

## Supplementary Information

Supplementary Material 1: Supplemetary Fig. 1. Distribution of the Propensity Score in the Treated and Control Groups. Kernel density estimates of the propensity score for women with a history of breast cancer (Treated) and those without such history (Control). Supplementary Fig. 2. Love plot of covariate balance before and after propensity score matching. Supplementary Table 1. Global Balance Diagnostics.

## Data Availability

No datasets were generated or analysed during the current study.

## References

[1] Jiang Y, Xing A, Hidru TH, Li J, Yang X, Chen S, et al. The association between arterial stiffness and cancer occurrence: data from Kailuan cohort study. Front Cardiovasc Med. 2023;10:1112047. 36937940 10.3389/fcvm.2023.1112047 PMC10014543

[2] Paterson DI, Wiebe N, Cheung WY, Mackey JR, Pituskin E, Reiman A, et al. Incident cardiovascular disease among adults with cancer: a population-based cohort study. JACC CardioOncol. 2022;4(1):85–94. 35492824 10.1016/j.jaccao.2022.01.100 PMC9040097

[3] Kawano T, Mackman N. Cancer patients and ischemic stroke. Thromb Res. 2024;237:155–62. 38603819 10.1016/j.thromres.2024.03.019

[4] Lee M, Meghan M, et al. Chronic kidney disease in cancer survivors. Adv Chronic Kidney Dis. 2021;28(5):469-476.e1. 35190113 10.1053/j.ackd.2021.10.007

[5] Melchiori R, et al. Cancer as a novel risk factor for major cardiovascular adverse events in secondary prevention. Int J Cardiol Cardiovasc Risk Prev. 2025;27:200501. 40978575 10.1016/j.ijcrp.2025.200501 PMC12445231

[6] Koric A, Chang CP, Mark B, Rowe K, Snyder J, Deshmukh VG, et al. Cardiovascular disease risk in long-term breast cancer survivors: a population-based cohort study. Cancer. 2022;128(14):2826–35. 35561317 10.1002/cncr.34224 PMC9991862

[7] Alarhabi AY, Mohamed MS, Ibrahim S, Hun TM, Musa KI, Yusof Z. Pulse wave velocity as a marker of severity of coronary artery disease. J Clin Hypertens. 2009;11(1):17–21. 10.1111/j.1751-7176.2008.00061.x PMC8673367 19125854

[8] Jae SY, Heffernan KS, Kurl S, Kunutsor SK, Laukkanen JA. Association between estimated pulse wave velocity and risk of stroke in middle-aged men. Int J Stroke. 2021;16(5):551–5. 33045935 10.1177/1747493020963762

[9] Townsend RR, Anderson AH, Chirinos JA, Feldman HI, Grunwald JE, Nessel L, et al. Association of pulse wave velocity with CKD progression and mortality: findings from the CRIC Study. Hypertension. 2018;71(6):1101–7. 29712736 10.1161/HYPERTENSIONAHA.117.10648 PMC6342478

[10] Ali S, Mullen KA. Challenges and opportunities for improving cardiovascular health in women with breast cancer: a review. Cardio-Oncol. 2025;11:72. 10.1186/s40959-025-00362-1 PMC12335184 40783746

[11] Grillo A, Moretti F, Scalise F, Faini A, Rovina M, Salvi L, et al. Comparison between invasive and non-invasive methods to evaluate aortic stiffness by pulse wave velocity. Artery Research. 2018;24:C92. 10.1016/j.artres.2018.10.101.

[12] Walser M, Schlichtiger J, Dalla-Pozza R, et al. Oscillometric pulse wave velocity estimated via the Mobil-O-Graph shows excellent accuracy in children, adolescents and young adults: an invasive validation study. J Hypertens. 2024;41(4):597–607. 10.1097/HJH.0000000000003374. 10.1097/HJH.0000000000003374 36723480

[13] Juhasz V, Drobni ZD, Quinaglia T, et al. Atorvastatin and aortic stiffness during anthracycline-based chemotherapy: a secondary analysis of a randomized clinical trial. JAMA Cardiol. 2026;11(1):68–76. 10.1001/jamacardio.2025.4548. 41205202 10.1001/jamacardio.2025.4548 PMC12596745

[14] Li J, Gao F, Cao F, Lv S, et al. Association of estimated pulse wave velocity with cardiovascular disease outcomes and all-cause death—a systematic review and meta-analysis. Front Cardiovasc Med. 2025;12:1641697. 10.3389/fcvm.2025.1641697. 41035700 10.3389/fcvm.2025.1641697 PMC12479400

[15] Kaboré EG, Macdonald C, Kaboré A, et al. Risk prediction models for cardiotoxicity of chemotherapy among patients with breast cancer: a systematic review. JAMA Netw Open. 2023;6(2):e230569. 36821108 10.1001/jamanetworkopen.2023.0569 PMC9951037

[16] Parr SK, Liang J, Schadler KL, et al. Anticancer therapy–related increases in arterial stiffness: a systematic review and meta-analysis. J Am Heart Assoc. 2020;9(14):e015598. 32648507 10.1161/JAHA.119.015598 PMC7660726

[17] Mohammadi Jouabadi S, Claringbould A, Danser AHJ, Stricker BH, Kavousi M, Roks AJM, Ahmadizar F. High-sensitivity C reactive protein mediates age-related vascular dysfunction: the Rotterdam study. Eur J Prev Cardiol. 2025:zwaf370. 10.1093/eurjpc/zwaf370. Epub ahead of print. PMID: 40561112. 10.1093/eurjpc/zwaf370 40561112

[18] Li Q, Zhang G, Li X, et al. Risk of cardiovascular disease among cancer survivors: systematic review and meta-analysis. EClinicalMedicine. 2025;84:103274. 40524799 10.1016/j.eclinm.2025.103274 PMC12169785

[19] Herzog MJ, Müller P, Lechner K, et al. Arterial stiffness and vascular aging: mechanisms, prevention, and therapy. Signal Transduct Target Ther. 2025;10(1):282. 40887468 10.1038/s41392-025-02346-0 PMC12399776

[20] Rios FJ, de Ciuceis C, Georgiopoulos G, et al. Mechanisms of vascular inflammation and potential therapeutic targets. Hypertension. 2024;81(6):1218–32. 38511317 10.1161/HYPERTENSIONAHA.123.22483

[21] Textor J, van der Zander B, Gilthorpe MS, Liskiewicz M, Ellison GT. Robust causal inference using directed acyclic graphs: the R package dagitty. Int J Epidemiol. 2016;45(6):1887–94. 28089956 10.1093/ije/dyw341

[22] Austin PC. An introduction to propensity score methods for reducing the effects of confounding in observational studies. Multivar Behav Res. 2011;46(3):399–424. 10.1080/00273171.2011.568786 PMC3144483 21818162

[23] Chaosuwannakit N, D’Agostino R Jr, Hamilton CA, Lane KS, Nian H, Jordan JH, et al. Aortic stiffness increases upon receipt of anthracycline chemotherapy. J Clin Oncol. 2010;28(1):166–72. 19901105 10.1200/JCO.2009.23.8527 PMC2799231

[24] Camilli M, Cipolla CM, Dent S, Minotti G, Cardinale DM. Anthracycline cardiotoxicity in adult cancer patients: *JACC* cardiooncology state-of-the-art review. JACC CardioOncol. 2024;6(5):655–77. 39479333 10.1016/j.jaccao.2024.07.016 PMC11520218

[25] Lefferts EC, Lee DC. Greater adherence to life’s essential 8 for cardiovascular health is associated with lower arterial stiffness in survivors of cancer. J Am Heart Assoc. 2024;13(12):e032886. 38842278 10.1161/JAHA.123.032886 PMC11255755

