# Supplementary material for: Arterial stiffness in long-term breast cancer survivors: a propensity score–matched analysis in primary prevention
Source: Cardiooncology. 2026 Mar 26;12:57. doi: 10.1186/s40959-026-00476-0 (PMC13141257; doi:10.1186/s40959-026-00476-0)
Supplement: Supplementary file 1 — Supplementary Material 1: Supplemetary Fig. 1. Distribution of the Propensity Score in the Treated and Control Groups. Kernel density estimates of the propensity score for women with a history of breast cancer (Treated) and those without such history (Control). Supplementary Fig. 2. Love plot of covariate balance before and after propensity score matching. Supplementary Table 1. Global Balance Diagnostics. [file 40959_2026_476_MOESM1_ESM.docx]

Supplementary material

**Supplemetary Figure 1**. Distribution of the Propensity Score in the Treated and Control Groups.

Kernel density estimates of the propensity score for women with a history of breast cancer (Treated) and those without such history (Control).


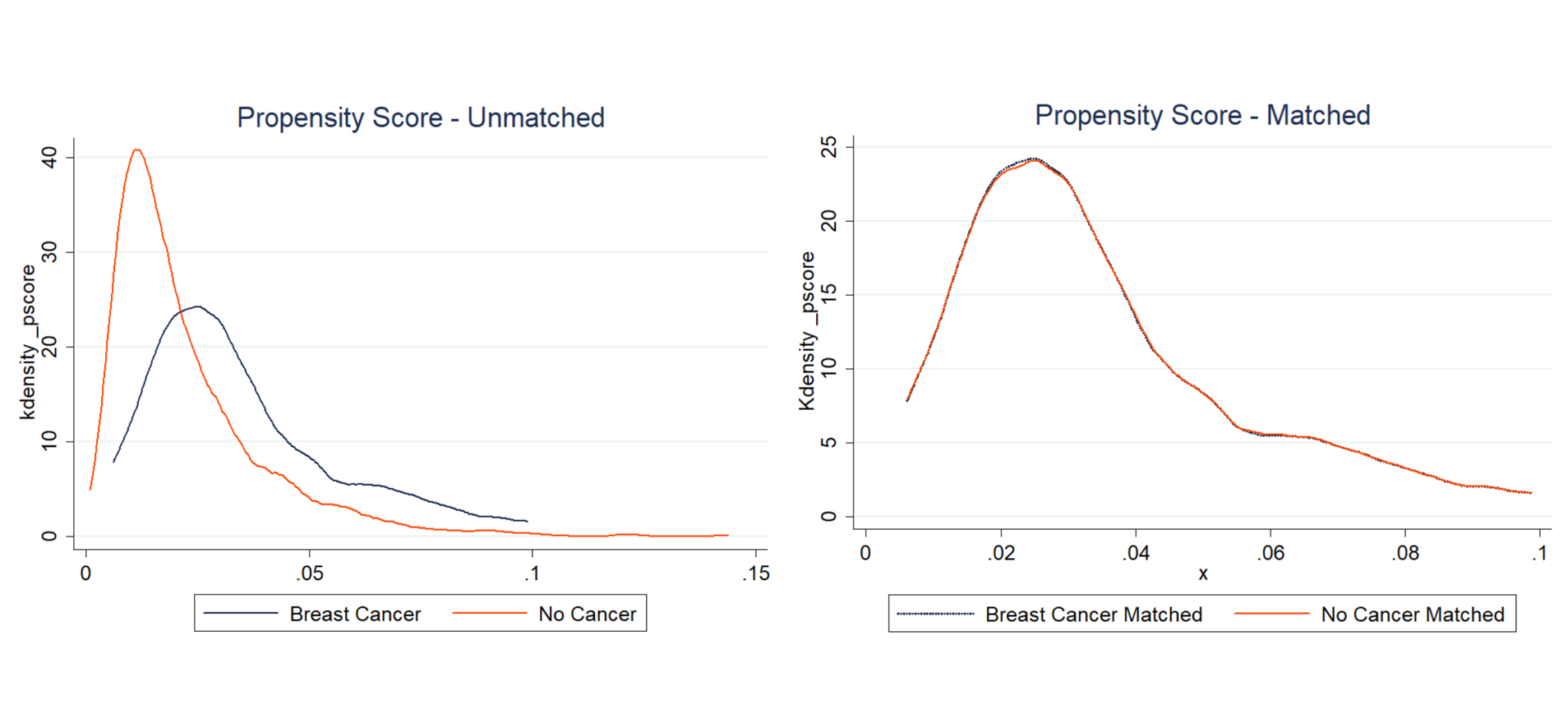


**Supplementary Figure 2. Love plot of covariate balance before and after propensity score matching.**


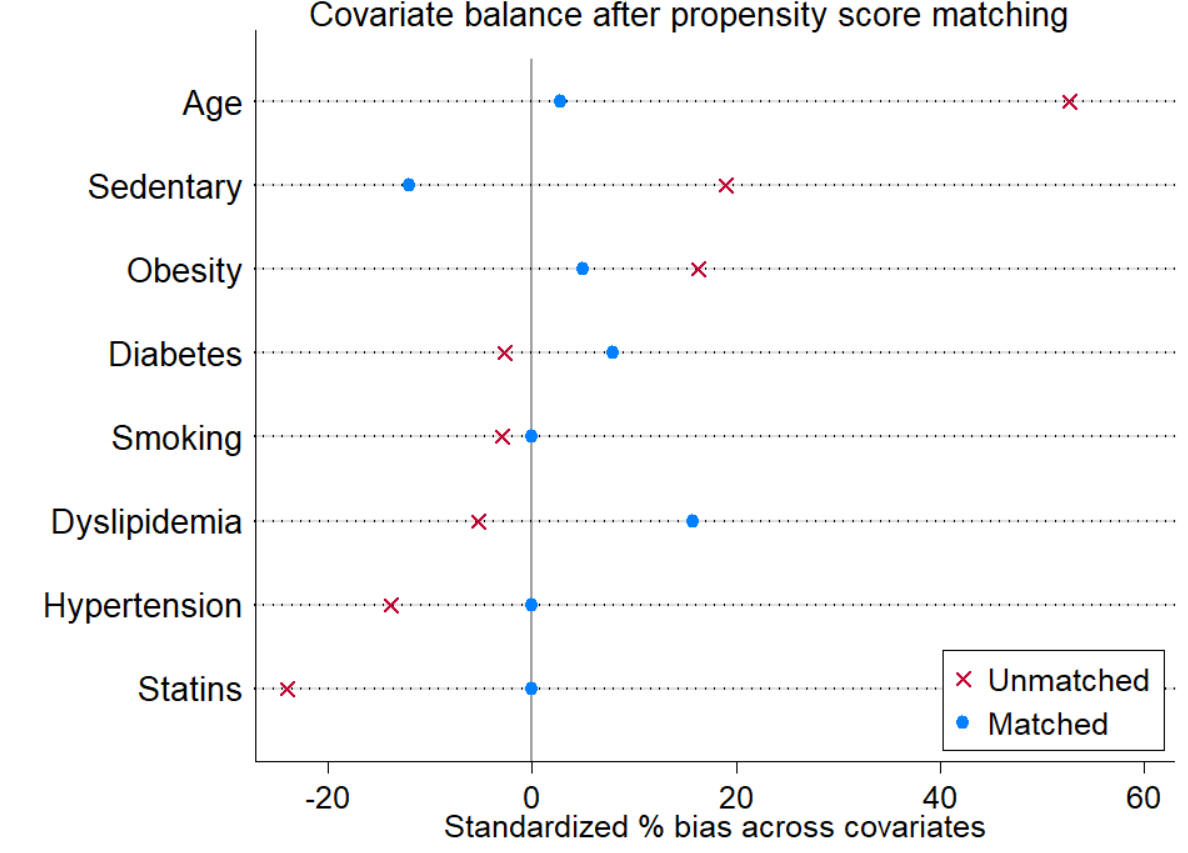


**Supplementary Table 1 Global Balance Diagnostics**

| **Metric** | **Before Matching** | **After Matching** |
| --- | --- | --- |
| Mean Standardized Bias | 17.1% | 5.4% |
| Median Standardized Bias | 15.0% | 3.8% |
| Pseudo R² | 0.056 | 0.014 |
| LR test p-value | <0.001 | 0.977 |
